# Shedding Light on “the Hole”: A Systematic Review and Meta-Analysis on Adverse Psychological Effects and Mortality Following Solitary Confinement in Correctional Settings

**DOI:** 10.3389/fpsyt.2020.00840

**Published:** 2020-08-19

**Authors:** Mimosa Luigi, Laura Dellazizzo, Charles-Édouard Giguère, Marie-Hélène Goulet, Alexandre Dumais

**Affiliations:** ^1^Research Center, Institut universitaire en santé mentale de Montréal, Montreal, QC, Canada; ^2^Departement of Psychiatry and Addictology, University of Montreal, Montreal, QC, Canada; ^3^Faculty of Nursing, University of Montreal, Montreal, QC, Canada; ^4^Institut national de psychiatrie légale Philippe-Pinel, Montreal, QC, Canada

**Keywords:** solitary confinement, segregation, inmates, symptom, mental health, mortality, meta-analysis, systematic review

## Abstract

**Objectives:**

To systematically review and meta-analyze the psychological effects and mortality rate in inmates having been exposed to solitary confinement in correctional settings.

**Methods:**

PubMed, PsycINFO, Web of Science, and Google Scholar were searched using keywords describing solitary confinement in combination with keywords for psychological or mortality outcomes. Eligible case-control studies for the systematic review met an operational definition for solitary confinement and evaluated outcomes after exposure to such confinement. Studies presenting statistical data which allowed to compute standardized mean differences for symptom scales or odds ratio for mortality were further meta-analyzed using random-effects models.

**Results:**

Systematic review identified 13 studies for inclusion, with a total sample of 382,440 inmates (23% having been exposed to solitary confinement). Higher quality evidence showed solitary confinement was associated with an increase in adverse psychological effects, self-harm, and mortality, especially by suicide. Meta-analysis of five studies (n = 4,517) showed a standardized mean difference of 0.45 for general psychological symptomatology, which increased to 0.51 upon outlier exclusion. Small to moderate significant effects were observed for mood, psychotic, and hostility symptoms specifically. In addition, meta-analysis of two mortality studies (n = 243,050) showed a trend for a moderate effect for mortality by any or unnatural causes (i.e., suicide, homicide, overdose, and accidents).

**Conclusions:**

Analyses showed that solitary confinement is associated with the psychological deterioration of inmates. This effect appears to be beyond that of general incarceration or presence of prior mental illness. Thus, solitary confinement may pose significant harm for inmates. Still, further studies are required to show that exposure to SC can increase risk of post-release death. Finally, add-on treatments and alternatives to solitary confinement that could alleviate the associated psychological harm are discussed.

## Introduction

As an umbrella term, solitary confinement (SC) subsides a range of denominations used in correctional settings, including but not limited to administrative or disciplinary segregation, protective custody, supermax, and restrictive housing (1). Although no universal definition exists for SC and there is considerable variability in policies, practices, and confinement conditions across facilities (1–3), SC generally refers to placement in restricted housing for upward 22–23 h per day, with heightened cell restrictions and security procedures (4). Apart from some exceptions, social contact with correctional staﬀ and other inmates is essentially eliminated. Inmates in SC are therefore accorded very little access to education, vocational training, visitation, recreation, and additional services that are available to the general inmate population. In this sense, mental health and medical services are even more limited (4, 5). Compared to general population housing, SC is also more than twice as costly (6).

Despite these costs, roughly 7% of inmates in the United States are placed in various forms of restrictive housing at any time (6, 7). Proponents of SC argue that the practice is necessary to maintain correctional safety, increase systemwide order, improve the behavior of violent and disruptive inmates, reduce gang inﬂuence, and improve the overall efficiency of correctional operations (4, 8). However, there is a shortage of empirical evidence demonstrating that SC truly accomplishes these aims (4). In fact, studies have shown that SC fails to reduce institutional misconduct in inmates (3, 9–13) and could even increase said misconduct (3, 14). SC has also been associated with an increase in subsequent recidivism or rule-breaking in community (15–17) and might therefore have adverse effects which outlive incarceration.

Exposure to these settings may even be associated with deleterious psychological effects. Whether it be known as “isolation sickness” or “security housing unit syndrome”, several scholars on SC have documented a set of debilitating mental health symptoms associated with residing in these environments (18, 19). Although there is an overrepresentation of inmates with mental illnesses in SC (2, 20), such settings have been reported to aﬀect inmates regardless of a pre-existing diagnosis (21). Hence, SC is not only particularly deleterious for inmates with existing mental health needs (22), but may bring forth new psychiatric symptoms and disorders, particularly stress-related disorders (adjustment, affective, and post-traumatic stress disorders) (23, 24). Early observations (21) were followed by case-studies (25–27) and a number of larger studies documenting adverse effects (19, 28). In short, secluded inmates have been observed to endure a range of harmful effects that vary in severity as they become progressively more anxious, depressed, irritable, confused, aggressive, and suicidal over time. This distress may be particularly potent when SC exposure is prolonged (29). Nevertheless, other studies have observed that if present, the negative effects of SC may dissipate with time (21, 30). Noteworthy, the methodological limits of previous literature (i.e., lack of control for confounders, reliance on qualitative accounts) have cast doubts on the negative effects of SC; some scholars even claiming that its effects are comparable to those of general incarceration (15). Beyond both these opposing perspectives, other experts have contended that the methodological limits of this literature are substantial and that meta-analytical investigation would allow to clarify the effects of SC (31).

Amidst this polarized literature, a first quantitative synthesis found administrative segregation specifically had a small to moderate association with worsened general mental health; with significant effects for mood/emotion (15). However, as recognized by experts during recent legal deliberations (32), this synthesis was flawed with some methodological issues. Certain details pertaining to the exclusion of articles and the design rating strategy were not included. Notably, the synthesis relied heavily on a single study (33) to generate 48% to 67% of effect sizes on psychological symptoms. Despite this study being overly criticized (34) in SC literature, it was rated among the study designs of higher quality. Finally, although reporting an association between self-harm and SC, suicidal behavior and mortality by suicide could not be investigated in relation to SC (15). Given these gaps and more recent publications with stronger methodology, a meta-analytical investigation is warranted to clarify the relationship between SC and psychological symptomatology and mortality outcomes. To assess the bulk of publications, both a systematic review and meta-analysis were carried out.

## Methods

### Search Strategy

A systematic review of literature was conducted in the electronic databases of PubMed, PsycINFO, Web of Science, and Google Scholar by ML and LD. The search included records from inception of databases until March 2020. No restrictions on geographical location were set. This search was carried out and reported in accordance with PRISMA recommendations (35) (see **Supplementary Table 1**). Text search and indexing terms (MeSH) defining SC (i.e., supermax, administrative segregation, disciplinary segregation, protective custody, restrictive housing, seclusion, maximum unit, special housing unit, security housing unit) were entered in combination with its potential health effects (i.e., mortality, suicide, anxiety, depression, anger, psychosis, hallucinations). Both categories of terms were entered in English and French. For the full electronic search strategy, please see **Supplementary Table 2**. Cross-referencing of all reviews and included articles was carried out by hand to identify any further publications. Authors were contacted for access to documents with restricted access and additional statistics to include in the meta-analysis. The records were compiled, and duplicates removed using EndNote X9. Title, abstract and full-text screening were carried out by ML and LD independently and verified by AD. This meta-analysis was registered on Figshare (10.6084/m9.figshare.12253358).

### Study Eligibility

Studies first underwent a full-text eligibility screening for inclusion in the systematic review. At this step, studies were included so long as they met five criteria:

1) The definition of SC followed our operational definition, that is, the isolation of an inmate from the general population within a cell where they spend most of their time, usually from 22 to 24 h a day (1, 4);
2) A comparison group of general population inmates not exposed to SC was included to ensure that the observed effects were above those of usual incarceration. All studies therefore consisted of case-control designs;
3) Inmates were incarcerated in a federal, state, provincial, or remand establishment;
4) A measure of psychological symptomatology, self-harm, or mortality was included, and
5) Effects of SC were not measured upon placement into SC, but rather after exposure to avoid measuring predictors of placement into SC.

For the meta-analyses, included studies met all the above criteria in addition to the following criterion:

6) Either symptoms were reported on a scale to compute a standardized mean difference (SMD) or data allowed calculation of odds ratio (OR) for mortality.

In all steps, studies in which inmates volunteered for placement into SC were excluded. Further, studies were not included in statistical analyses if they presented significant methodological issues (i.e., reanalysis of the same cohort, insufficient data to compute effects) despite having contacted authors for further data. Disagreements on the inclusion of studies were resolved by group discussion between ML, LD, CEG, and AD.

### Data Extraction

Data was extracted independently and double-checked by ML and LD to insure consistent reporting within a standardized extraction form. Effect sizes were reported with 95% confidence intervals (CI) in the form of SMD for symptoms and OR for mortality. Quality of all studies selected for review and meta-analysis was assessed by ML, LD, and AD following a set of criteria based on the GRADE Checklist for observational studies (36–39) (see **Supplementary Table 3**). Selected studies were classified as High, Moderate, Low, or Very Low quality. Evidence graded as high quality was provided by a study with prospective design, controlled for confounding factors (e.g., time since incarcerated, previous mental illness, gender), used a reliable and standardized outcome measure and included a large total sample. Evidence graded as very low quality was provided by a study with retrospective design, no control for confounders, use of a non-standardized scale or self-report without clinician evaluation and comprised of a small sample. Lastly, upon consensus between authors, we attempted to decrease the effect of certain limits of the Colorado Report (33) by only considering symptoms in the first three months after “baseline” and therefore include the effect of what was mostly considered disciplinary segregation. Indeed, both groups awaited their trial in disciplinary segregation and the average time until transfer to administrative segregation for the “exposed group” was three months after baseline. Consequently, during the three initial months considered here, the control group had been returned to general population, whereas the exposed group progressively transferred from disciplinary to administrative segregation as beds became available (33).

### Analyses

The main outcomes were symptoms relating to mental health state following SC and mortality among post-release inmates exposed or not exposed to SC. These outcomes were first described in a systematic review to consider articles that could not be included in meta-analysis for statistical reasons (lack of symptom scale or statistical analysis). Secondly, meta-analyses were carried out on the studies allowing for computation of effect sizes for (i) all symptoms combined to achieve a measure of general symptomatology severity, (ii) specific categories of psychiatric symptoms (mood, psychotic, and hostile/aggressive symptoms), and (iii) mortality outcomes. Based on previous research (15, 21) and subscales for common psychiatric scales (40), symptom categories consisted of mood symptoms (i.e., depression and anxiety), psychotic symptoms (i.e., psychoticism and thought disorder), and aggressivity/hostility (i.e., animosity, property destruction, verbal or physical aggression toward others). Mortality was considered for all causes and unnatural causes specifically (suicide, homicides, overdoses, and accidents). Data were entered into an electronic database and analyzed with a quantitative meta-analytical approach using R version 3.6.2 (R core team) and the metafor package (41).

For each outcome, data was pulled from selected studies to generate a SMD on the symptom scales or an OR for mortality. As generally evaluated in observational research, SMD were classified as *small* (0.2), *medium* (0.5), and *large* (≥0.8) (42). ORs were classified as follows: *small* (OR = 1.0–1.5), *moderate* (OR = 1.6–2.5), *strong* (OR = 2.6–9.9), and *very strong* (OR ≥ 10.0) (43). Both outcomes were coded so that a positive value represented a poorer outcome for inmates exposed to SC in comparison to the general inmate population. Multiple effect sizes per article were considered when this was possible to account for the whole range of symptoms or mortality causes investigated. We therefore employed a precautionary statistical approach, adding a variance term for each article from which multiple effect sizes were derived.

Heterogeneity among study point estimates was assessed with Q statistics (44) and the magnitude of heterogeneity with the *I^2^* index (45). Publication bias was examined by way of funnel plot and Egger’s test (46, 47). In the event of a significant Egger’s test, although it is not meant to reflect the strength of effect sizes, Rosenthal’s fail-safe test was also reported to more clearly illustrate possible publication bias (48, 49). Outliers were defined as data points two or more standard deviations above or below the effect size mean. Because significant heterogeneity was observed (see results), we employed random-effects models that are more conservative than fixed-effects models and appear to better address heterogeneity between studies and samples (50). Moreover, an exploratory sub-analysis was carried out by removing a study by O’Keefe et al. (51) as the sample was the only one entirely composed of inmates who suffered from a known mental illness.

## Results

**Figure 1:**
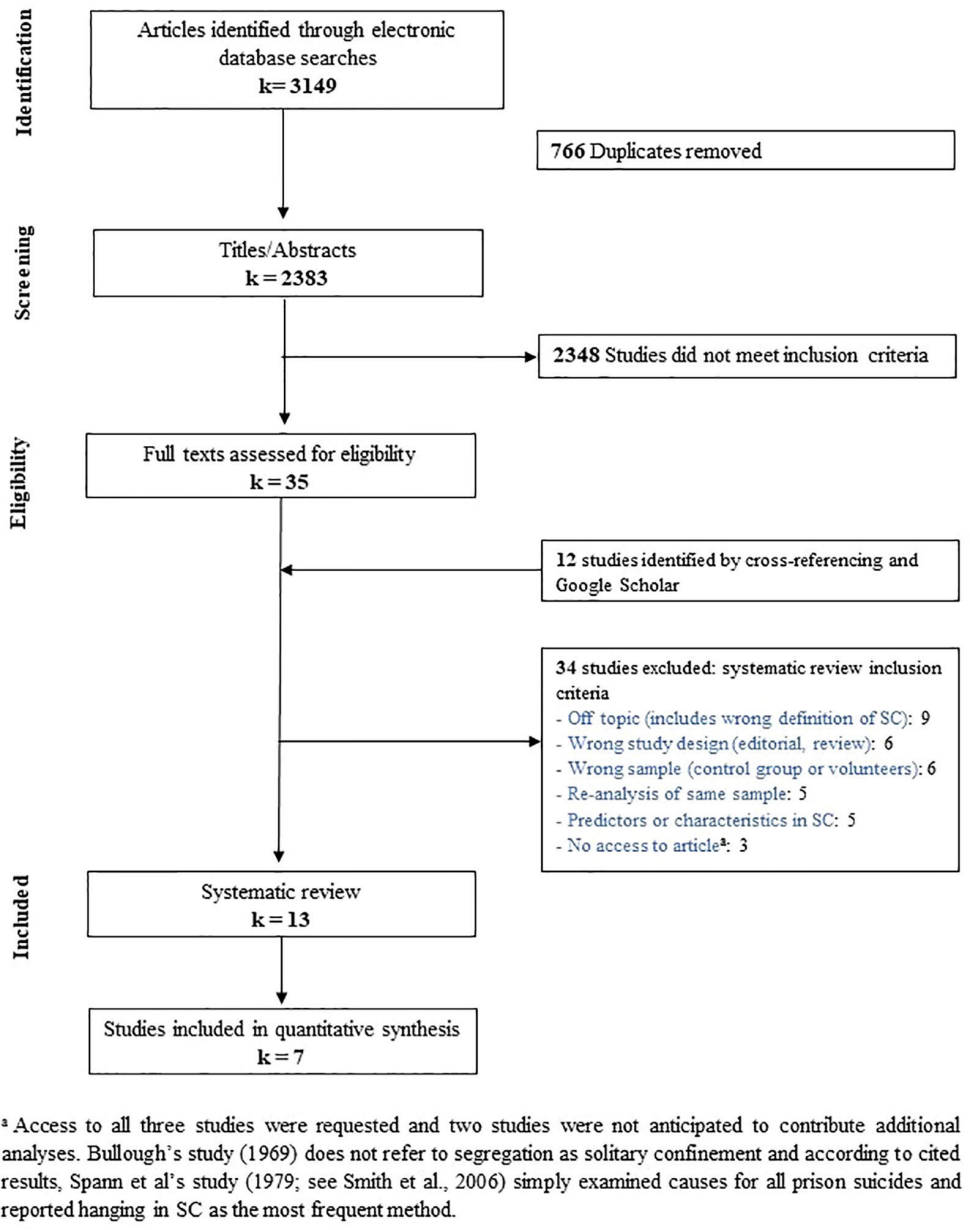
Flowchart of the search strategy to identify the 13 studies included in systematic review and 7 studies in meta-analysis.

After removing duplicates, the literature search for the mental health and mortality impacts of SC identified 2,383 records to be screened for eligibility. Among these, 35 full texts were assessed, and 12 were identified through cross-referencing and Google Scholar. From these 47 potential articles, 13 were ultimately included in the systematic review and seven out of 13 were included in the meta-analysis. Please refer to **Figure 1** for a breakdown of our exclusion process and **Supplementary Table 3** for details on all included studies. Taken together, all 13 studies regrouped 382,440 inmates (roughly 23% in SC), originating mainly from the United States (96%); inmates in remaining studies (4%) originated from Denmark and Canada.

### Systematic Review

Firstly, the effects of symptomatology were separated into mood, psychotic, and hostile/aggressive symptoms. Eight studies reported effects relating to depressive symptoms. The evidence of six of these studies was ranked as moderate and two as low quality. Regarding depression and dysthymia, three studies with moderate quality evidence found higher scores in inmates having been exposed to SC (52–54). However, low quality evidence from two other studies (30, 55) and one study with moderate quality evidence (51) did not observe differing depression scores between groups. Lower quality evidence was provided by studies which controlled for minimal (race and ethnicity) or no confounders and instructed inmates to self-report psychological symptoms (30, 55). Moreover, two prospective studies examined depressive symptoms longitudinally. Both used semi-structured interviews to evaluate psychological outcomes and evidence was rated of moderate quality. In their study with follow-ups up to two months into SC exposure, Zinger and Wichmann (54) observed overall improvements. In Andersen et al.’s study (56), follow-ups were reported up to 3 months after initial SC transfer, although there was attrition in the SC cohort across time. The authors observed that SC inmates remained depressed while non-SC inmates improved through follow-up. However, no confounders were controlled for in both studies and comparison groups were not matched on demographic characteristics.

For stress- or anxiety-related disorders, four studies presenting moderate quality evidence reported higher anxiety (52–54) and post-traumatic stress symptoms (23, 52) in SC inmates. Zinger and Wichmann (54) found higher mean anxiety scores for SC inmates, yet similar improvement across follow-up for both groups (SC and non-SC inmates). Andersen et al. (56) found the anxiety scores of SC inmates remained high while those in general population improved over follow-up. Finally, three studies did not observe different post-exposure anxiety scores, two of low (30, 55) and one of moderate (51) quality.

Four studies considered psychotic symptoms. Two studies provided moderate quality evidence that found SC inmates showed elevated psychotic symptoms (51, 53). The main strength common to both studies was the use of the *Brief Psychiatric Symptom Scale* by a clinician. However, one study of moderate quality (52) and one study of low quality (55) did not find differing levels of psychotic symptoms between groups.

Regarding hostility, three studies found worse outcomes in SC groups. Among these, evidence in two studies was evaluated as moderate quality (51, 53) and one of low quality (55). Only one study in which inmates self-reported symptoms and no statistical controls were included (30) found no difference in hostility scores between groups. Still, this study also found longer time in SC was associated with a significant increase in hostility. From a longitudinal perspective, Zinger and Wichmann (54) found no significant difference in aggression between groups of SC and non-SC inmates across follow-up.

Secondly, five studies examined self-harm (33, 57, 58) and suicidal ideation/behavior (17, 33, 59). The evidence provided by these studies was rated as high (17), moderate (57–59) and low quality (33). From moderate quality evidence, SC inmates were found to be between 1.56 (57) and 6.89 (58) times more at risk of self-harm. Both studies were controlled for age and mental health history, along with their own lists of confounding factors (ethnicity, education, length of incarceration, prior misconducts, prior violent offense). Moreover, one study supplied low-quality evidence for self-harming ideation and behavior in inmates with mental illnesses (33). Ten inmates with mental illnesses in SC (20%) reported such events compared to only two (8%) in general inmate population. This study was rated as low quality because the symptom scale was not administered by a clinician, no statistical comparison was performed and therefore no confounders were considered.

Regarding suicide, one study (17) found inmates exposed to SC were 1.78 times more likely to die by suicide within a year (adjusted hazard ratio = 1.78 [1.19–2.67]). This evidence was evaluated as high quality mainly because of control for numerous confounders (age ranges, number of prior incarcerations, drug or violence-related convictions, mental health treatment recommended or received, quartiles of number of days served during recent sentence, time-fixed sex, and race) and a large sample size (n = 229,274). A second study (59) found SC inmates were 2.34 times more likely to die by unnatural causes within 5-year post-release (59) (adjusted hazard ratio = 2.34 [1·53–3·59]); with unnatural deaths referring to fatal self-harm, accidents and violence. This study controlled for multiple confounders although not all (i.e., no control for mental health status) and did not report a separate analysis for death by suicide. This evidence was rated of moderate quality.

Thirdly, both high (17) and moderate (59) graded evidence on mortality indicated increased death in community from all causes (OR = 1.24 and 1.97, respectively). High quality evidence from one of these studies also showed increased death by opioid overdose (17) (OR = 1.27) and homicide (17) (OR = 1.54).

### Meta-Analysis

For meta-analyses, the selected studies amounted to a total sample of 4,517 individuals for symptoms (k = 5) and 243,050 for mortality (k = 2) analyses; all were presently or formerly incarcerated. Overall, 32% had been exposed to SC during the study’s evaluation period. All but two studies originating from Canada (54) and Denmark (59) were from the United States. Evidence provided by these seven studies were evaluated as high (17), moderate (51, 52, 54, 59), and low (33, 55). As for study design, five studies were longitudinal (two prospective and three retrospective) and two were cross-sectional.

#### Psychological Symptoms

**Figure 2:**
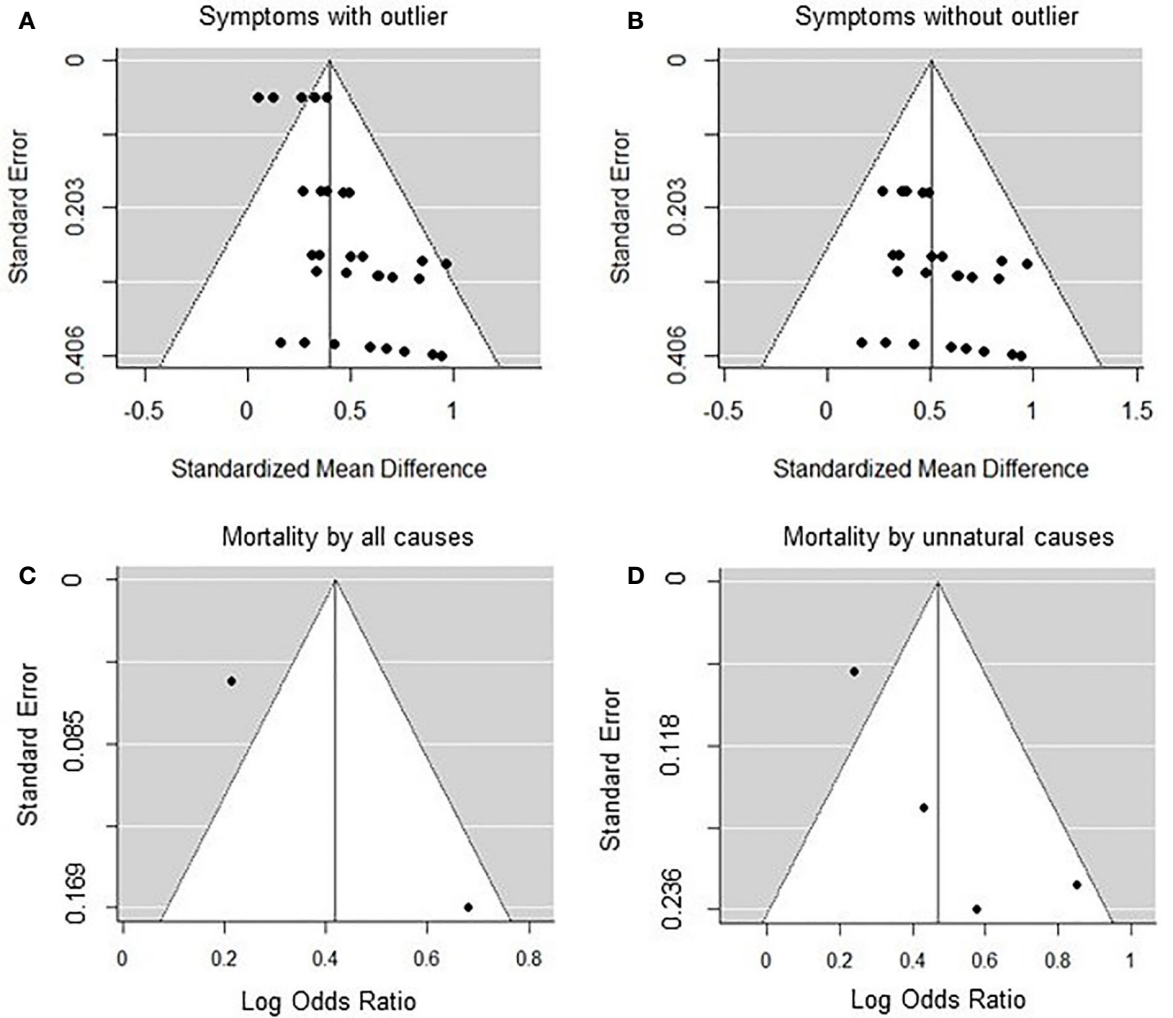
Funnel plots of the meta-analyses for solitary confinement and psychological symptoms with **(A)** or without **(B)** outlier and mortality by all **(C)** or unnatural **(D)** causes.

Symptoms were either evaluated as part of correctional clinical evaluations (51), by trained graduate students (33, 54) or self-reported by inmates (52, 55). The database was characterised by high heterogeneity (Q = 68.32, *p* < 0.001, *I^2^* = 63.58%). Removal of one study (51) on mentally ill inmates rendered the database without significant heterogeneity (Q = 14.3, *p* = 0.94, *I^2^* < 0.001%) and no visually apparent publication bias (see **Figure 2**). Publication bias was also examined by conducting an Egger’s test, which was significant (p = 0.023). However, Rosenthal’s fail-safe test showed that 820 negative studies would be needed to invalidate significant results presented below.

**Figure 3:**
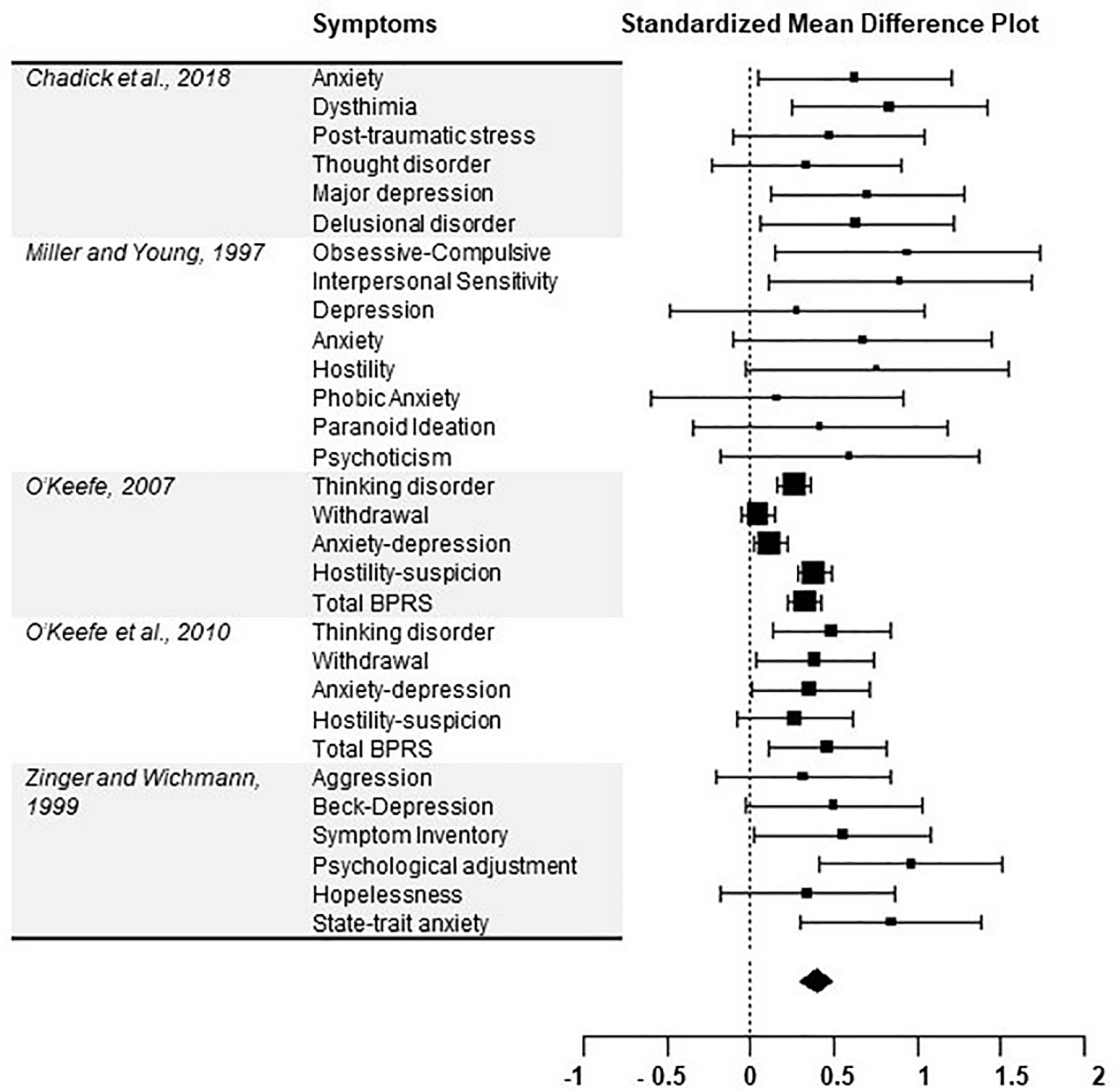
Forest plot of the association between inmate exposure to solitary confinement and mean difference for any mental health symptom.

The SMD on mental health scales from the pooled five studies was 0.45 (CI 95%= 0.29–0.61, *p* < 0.001) (see **Figure 3**). This analysis was repeated after removing the same study (51) as above, yielding a SMD of 0.51 (CI 95% = 0.39–0.63, *p* < 0.001). Furthermore, three sub-analyses for specific symptom categories were carried out on all five identified articles (see **Table 1** for descriptive statistics). Scores on mood (anxiety/depression) scales varied significantly for SC inmates, with an SMD of 0.41 (CI 95% = 0.19–0.64, *p* < 0.001). Psychotic symptoms also differed significantly between SC and general population inmates, with an SMD of 0.35 (CI 95% = 0.18–0.52, *p* < 0.001). Concerning aggressivity or hostility symptoms, the SMD between both groups was 0.38 (CI 95% = 0.29–0.47, *p* < 0.001).

**Table 1:** Descriptive statistics of sub-analyses.

Symptom Category	Number of included effect sizes	N	Effect size	95% CI	Heterogeneity		
					Q	*I^2^*	*p-value*
*Mood (anxiety and depression)*	17	4,517	0.41*	0.19–0.64	37.71	65.46%	*p = 0.00*
*Psychotic symptoms*	5	4,457	0.35*	0.18–0.52	3.49	21.12%	*p = 0.48*
*Aggressivity (hostility and aggression)*	4	4,469	0.38*	0.29–0.47	1.36	0%	*p = 0.72*
**Mortality outcome**	**Number of included effect sizes**	**N**	**Effect size**	**95% CI**	**Q**	***I^2^***	***p-value***
*All causes*	2	243,050	1.52	0.97–2.39	6.88	85.47%	*p = 0.01*
*Unatural causes*	4	243,050	1.70	0.98–2.94	9.16	65.96%	*p = 0.03*

*Significant difference between groups exposed or not to solitary confinement, p < 0.001.

#### Mortality

Effect sizes for both all causes (Q = 6.88, *p* < 0.05, *I^2^* = 85.47%), and unnatural causes (Q = 9.16, *p* < 0.05, *I^2^* = 65.96%) showed high heterogeneity. No publication bias was observed (see **Figure 2**). Egger’s test was not valid given the inclusion of too few (two to four) effect sizes per mortality analysis.

The risk of mortality from all causes was not significantly different between groups (OR = 1.52, CI 95% = 0.97–2.39, *p* = 0.069), nor was mortality from unnatural causes (OR = 1.70, CI 95% = 0.98–2.94, *p* = 0.06).

## Discussion

Our systematic review and meta-analysis were conducted to synthesize literature concerning the effects of SC on mental health symptoms as well as on mortality. Despite opposing views within the field, our results showed a significant moderate association between SC and increased symptomatology. No publication bias was observed, given that the Rosenthal fail-safe *k* was an excessively large number considering the paucity of identified studies for this and previous synthesis (15). Trends were present for moderate associations between SC and mortality in community (OR = 1.52 and 1.70). Higher quality studies from the systematic review also showed SC was related to deleterious effects with regards to mood symptoms, PTSD-related outcomes, psychotic experiences, hostility, self-injurious behavior, and mortality.

More specifically, SC was significantly related to increased mood symptomatology (SMD = 0.41), which is consistent with a prior quantitative synthesis (15). Recent investigations have related depression in inmates housed in the general population with a lack of social support and as a proxy, not receiving visits (60, 61). SC further restricts visits and eliminates opportunities to foster social connections by design, thereby enabling a potential increase of mood symptomatology. Moreover, our meta-analysis confirmed a small to moderate association with increased psychotic and aggressive/hostile symptoms. This is in contrast to prior synthesis which found nonsignificant associations between SC and such symptoms (15). This difference may be mainly attributable to the fact that our analyses gave the Colorado study (33) a weight equivalent to other studies, one of the major limitations that has been attributed to the prior synthesis (18, 34). Notably, our findings showing that SC relates to increased hostility are consistent with theories suggesting SC leads to feelings of unjust treatment, frustration, and rage (28). This state could partly explain why SC does not successfully reduce violent misconduct (3, 10, 12, 13) or may increase post-release recidivism (15, 17). Lastly, the significant association between an increase in psychotic symptoms and SC coincides with qualitative accounts of reactive psychotic symptoms following SC (21, 62, 63). Although substance use withdrawal could explain part of this association (64), psychosis has also been linked to restricted visitation in prison (60) and social isolation more broadly (65, 66). Still, it should not be interpreted that SC inmates develop well-formed psychotic disorders per se. A study on the incidence rate of psychotic disorders as a result of SC suggests that developing such disorders is rare (24). Moreover, it is possible that psychotic experiences do not persist long after SC (21).

More broadly, meta-analysis found a moderate overall association between SC and psychological symptomatology, which is consistent with prior non systematic reviews (4, 18, 19, 21). Above the explained mechanisms, SC comprises other core components that could generally result in such effects. Firstly, although issues of continuity in mental health care plague all correctional settings, these effects are accentuated in SC (15, 67). Indeed, psychiatric treatment within SC is usually limited to the administration of medicine and short and infrequent cell-front visits (*cell-front therapy*) (22, 29). Such obstructed access to inmates would make monitoring of psychological deterioration difficult and possibly under detected. From the observed self-harm in SC and the finding that inmates commit suicide in prison despite increased monitoring (68), it seems evident that simple monitoring in SC is not sufficient and that psychological support is required. Secondly, the design of SC generally denies inmates vocational programming, regular and unobstructed visitation, or opportunities for pro-social interactions; all of which are necessary for inmates’ mental health in prison and their successful release into community (69, 70). As a third factor, SC conditions may create a specific “culture of harm” among correctional staff (71) and many litigations have shed light on physical abuse, excessive use of restraints, and mistreatment of mentally ill inmates in SC (i.e., *Madrid vs Gomez*, *1995*, *Ruiz vs Johnson*, *1999* and *Valdes vs Crosby*, *2006*). Inmates exposed to SC may be as troubled by the negative staff attitudes as social isolation itself (30). Fourthly, as an alternative explanation for the effects of SC, it has been contended that the most distressed inmates are preferentially placed into SC and drive the association with mental deterioration. Indeed, prior mental illness is an important predictor of placement into SC (72). However, in our meta-analysis, the association between psychological deterioration and SC exposure grew even stronger when removing a sample entirely composed of inmates with prior mental illnesses (51). This, along with controlled studies (17, 23, 52, 57, 58), indicates that inmates with a known mental illness are not driving the entirety of the association between SC and psychological distress. Lastly, some studies observed that whereas general population inmates improved over time on measures of psychiatric symptoms, SC inmates remained stable (52, 56). If most inmates are indeed improving with time (54), stability in SC could be viewed as its own negative outcome. Given the apparent distress associated with SC, such observations should not impede putting in place relevant precautionary programming.

Finally, meta-analysis showed a trend toward moderate associations between SC and increased mortality by unnatural causes (suicide, opioid overdose, homicide, accidents) or all causes (unnatural and natural death). Given that both individual studies found a significant increase in mortality after SC in large samples [229,274 (17) and 13,776 (59)], it is likely our trend toward significance would become significant upon inclusion of future studies. Both these studies controlled for pre-placement characteristics (such as prior criminality and mental illness) and still found increased risk of mortality for inmates exposed to SC, suggesting these effects go beyond initial vulnerabilities. Findings included increased unnatural death [hazard ratio of 2·342 (59)] and death by suicide [hazard ratio of 1.78 (17)]. Our meta-analysis reported an increase in depression and hostility following SC which relate to higher risk of suicidal behavior (73). Additionally, one study found SC was associated with increased death by opioid overdose specifically (17), further suggesting a lack of psychiatric treatment for this sub-group. Moreover, homicide was also a cause of death included in the increase of unnatural deaths associated with SC in a prior study (59). Finally, the second mortality study found a significant increase for homicide specifically (hazard ratio of 1.54) (17). Because exposure to SC also seems to increase the risk of committing violence post-release (74), it is plausible that releasees also could be at risk of mortality in such altercations. Still, this is the first meta-analytical investigation of mortality following SC and more than two studies are required to further evaluate mortality outcomes.

To alleviate the psychological harms associated with SC, multiple steps of SC exposure can be targeted for programming and alternatives. The Vera Institute of Justice’s “Safe Alternatives” initiative has set out to provide reviews and evaluations on changes in the SC model which are emerging throughout American states (1, 75). Alternatives include, for instance, the use of mental health units for the treatment of inmates with known mental illnesses in a secure setting, which may prevent the placement of particularly vulnerable populations in SC (76). For example, the CAPS program in the state of New-York has been shown to reduce self-harm and injury in mentally ill inmates compared to restrictive housing (77). In this program, inmates are encouraged to spend a maximum amount of time out of their cell in order to attend multiple program (individual or group) and therapy sessions (78). Louisiana and North-Carolina have also begun to implement such mental health units as an alternative to SC (79, 80). Alternative units for all inmates intended for transfer into SC have also been put in place, offering increased programming related to inmates’ correctional plan and increased time out of cell (78, 80). Even when inmates are placed in more conventional SC units, the lack of access to already established vocational and re-entry programs should be revised to offer SC inmates a reintegration potential similar to that of the general inmate population (79). Further, periodic revision of cases should be conducted to safely transition inmates back to general inmate population at the earliest. With this policy, correctional and mental health staff should conjointly judge when release is appropriate (81). Such periodic evaluation of SC placement could help safeguard against the observed increase in self-harming behaviors. The harm of prolonged isolation on the mental health of inmates and the need for a cap on the maximum number of days in SC has been widely recognised (82, 83). Moreover, when transitioning out of SC, some correctional facilities have offered peer-mentor accompaniment or dormitory transition units with increased programming (79). These initiatives gradually allow inmates to become re-accustomed to frequent social contact, preventing them from being overwhelmed and socially withdrawing (28). Such “step-down programs” also offer a gradual increase in behavioral interventions and incentives (such as out-of-cell time) to help inmates successfully prepare for general inmate population and release into community (81). Finally, upon release, intensive case-management of all releasees or forensic assertive community treatment for inmates with lasting mental illness could also reduce subsequent criminal justice involvement and hospitalizations in releasees (84).

Our study serves as a critical and up-to-date appraisal of the literature on the effects of SC. Still, methodological limits must be considered. Our systematic review highlighted the variability in study and statistical designs. Use of meta-analytical investigation partly enabled to correct for these differences, but more large studies with statistical control and/or control groups matched on pre-placement differences may ensure robustness of future work and refine our knowledge of the effects of SC. Moreover, as reported in **Figure 3**, multiple scale items were grouped into clinically relevant categories (i.e., mood, aggressivity, and psychotic symptoms). This approach was used because studies utilized different scales for psychiatric symptoms, which might have yielded some heterogeneity. However, the percentage of variation across studies was not significant for two constructs (aggressivity and psychotic symptoms) and we employed random-effects models for all meta-analyses to address heterogeneity between studies and samples. Secondly, data on length of SC exposure was not systematically available. Future studies should examine “dosage” of SC to better inform recommendations on how long inmates could remain in SC before experiencing significant harm. Thirdly, it is possible that cross-sectional or retrospective designs, such as those used in most studies included, do not account for the loss of inmates so adversely affected by SC that they necessitate transfer out of this housing. As such, studies may have underestimated the distress brought on by SC. As the number of studies eligible for meta-analysis of psychiatric symptoms was low, further studies are needed to adequately compare if observed effects on mental health differ by study design. Lastly, institutions and jurisdictions may vary in the conditions in which SC inmates are held. Degree of social isolation, level of privileges, availability of programming, and extent of a “culture of harm” could all moderate the effects of SC (18). This variation could explain the observed heterogeneity in our sample. Future studies should thus report specific conditions to facilitate synthesis of literature.

In conclusion, our systematic review and meta-analysis found significant associations between SC and general mental health, mood, psychotic, and hostility symptoms. A trend was observed for relating SC to later death in community by any or unnatural causes. Furthermore, our review found an increased risk for self-harm. The association between SC and increased general psychological symptomatology does not seem driven by inmates with prior mental illnesses. Our findings support criticisms that SC prevents the rehabilitation of inmates compared to general incarceration but also that the practice may cause significant harm. More studies investigating causality are needed to confirm the mechanisms behind these effects and to evaluate risk of mortality post-release. Our findings highlight the cost of SC on inmates and the need to re-consider the policies behind SC and how it is delivered. The literature proposes certain add-on treatments or alternatives to reduce the unintended consequences of SC, but these will need to be rigorously evaluated to examine their effectiveness.

## Data Availability Statement

The original contributions presented in the study are included in the article/supplementary material; further inquiries can be directed to the corresponding author.

## Author Contributions

ML, LD, and AD were involved in the concept and design of the manuscript. ML and LD conducted the literature search and extraction. ML, LD, C-ÉG, MH-G, and AD contributed to the analysis and/or interpretation of data. ML drafted the manuscript. ML, LD, C-ÉG, MH-G, and AD were involved in the critical revision of the manuscript for intellectual content.

## Funding

No specific funding was awarded for this research. ML is holder of a master’s scholarship from the Fonds de Recherche du Québec en Santé and LD is holder of a doctoral scholarship from the Fonds de Recherche du Québec en Santé. AD is holder of a Junior 2 salary award from the Fonds de Recherche du Québec en Santé.

## Conflict of Interest

The authors declare that the research was conducted in the absence of any commercial or financial relationships that could be construed as a potential conflict of interest.
