# Supplementary material for: Shedding Light on “the Hole”: A Systematic Review and Meta-Analysis on Adverse Psychological Effects and Mortality Following Solitary Confinement in Correctional Settings
Source: Front Psychiatry. 2020 Aug 19;11:840. doi: 10.3389/fpsyt.2020.00840 (PMC7468496; doi:10.3389/fpsyt.2020.00840)
Supplement: Supplementary file 1 [file Table_1.docx]

Supplementary Material

# Supplementary Figures and Tables

| **Supplementary Table. 1** PRISMA Checklist | | | |
| --- | --- | --- | --- |
| **Section/topic** | **#** | **Checklist item** | **Reported on page #** |
| **TITLE** | | |  |
| Title | 1 | Identify the report as a systematic review, meta-analysis, or both. | 1 |
| **ABSTRACT** | | |  |
| Structured summary | 2 | Provide a structured summary including, as applicable: background; objectives; data sources; study eligibility criteria, participants, and interventions; study appraisal and synthesis methods; results; limitations; conclusions and implications of key findings; systematic review registration number. | 1 |
| **INTRODUCTION** | | |  |
| Rationale | 3 | Describe the rationale for the review in the context of what is already known. | 2 |
| Objectives | 4 | Provide an explicit statement of questions being addressed with reference to participants, interventions, comparisons, outcomes, and study design (PICOS). | 2 |
| **METHODS** | | |  |
| Protocol and registration | 5 | Indicate if a review protocol exists, if and where it can be accessed (e.g., Web address), and, if available, provide registration information including registration number. | 3 |
| Eligibility criteria | 6 | Specify study characteristics (e.g., PICOS, length of follow-up) and report characteristics (e.g., years considered, language, publication status) used as criteria for eligibility, giving rationale. | 3 |
| Information sources | 7 | Describe all information sources (e.g., databases with dates of coverage, contact with study authors to identify additional studies) in the search and date last searched. | 2 |
| Search | 8 | Present full electronic search strategy for at least one database, including any limits used, such that it could be repeated. | 2-3  Supp. Table 2 |
| Study selection | 9 | State the process for selecting studies (i.e., screening, eligibility, included in systematic review, and, if applicable, included in the meta-analysis). | 3  Figure 1 |
| Data collection process | 10 | Describe method of data extraction from reports (e.g., piloted forms, independently, in duplicate) and any processes for obtaining and confirming data from investigators. | 3 |
| Data items | 11 | List and define all variables for which data were sought (e.g., PICOS, funding sources) and any assumptions and simplifications made. | 3 |
| Risk of bias in individual studies | 12 | Describe methods used for assessing risk of bias of individual studies (including specification of whether this was done at the study or outcome level), and how this information is to be used in any data synthesis. | - |
| Summary measures | 13 | State the principal summary measures (e.g., risk ratio, difference in means). | 3 |
| Synthesis of results | 14 | Describe the methods of handling data and combining results of studies, if done, including measures of consistency (e.g., I^2^) for each meta-analysis. | 3 |

Page 2 of 2

| **Section/topic** | **#** | **Checklist item** | **Reported on page #** |
| --- | --- | --- | --- |
| Risk of bias across studies | 15 | Specify any assessment of risk of bias that may affect the cumulative evidence (e.g., publication bias, selective reporting within studies). | 4 |
| Additional analyses | 16 | Describe methods of additional analyses (e.g., sensitivity or subgroup analyses, meta-regression), if done, indicating which were pre-specified. | 3 |
| **RESULTS** | | |  |
| Study selection | 17 | Give numbers of studies screened, assessed for eligibility, and included in the review, with reasons for exclusions at each stage, ideally with a flow diagram. | Figure 1 |
| Study characteristics | 18 | For each study, present characteristics for which data were extracted (e.g., study size, PICOS, follow-up period) and provide the citations. | Supp. Table 3 |
| Risk of bias within studies | 19 | Present data on risk of bias of each study and, if available, any outcome level assessment (see item 12). | - |
| Results of individual studies | 20 | For all outcomes considered (benefits or harms), present, for each study: (a) simple summary data for each intervention group (b) effect estimates and confidence intervals, ideally with a forest plot. | 4-5  Figure 3 |
| Synthesis of results | 21 | Present results of each meta-analysis done, including confidence intervals and measures of consistency. | 6 |
| Risk of bias across studies | 22 | Present results of any assessment of risk of bias across studies (see Item 15). | 6 |
| Additional analysis | 23 | Give results of additional analyses, if done (e.g., sensitivity or subgroup analyses, meta-regression [see Item 16]). | 6,  Table 1 |
| **DISCUSSION** | | |  |
| Summary of evidence | 24 | Summarize the main findings including the strength of evidence for each main outcome; consider their relevance to key groups (e.g., healthcare providers, users, and policy makers). | 6 |
| Limitations | 25 | Discuss limitations at study and outcome level (e.g., risk of bias), and at review-level (e.g., incomplete retrieval of identified research, reporting bias). | 9 |
| Conclusions | 26 | Provide a general interpretation of the results in the context of other evidence, and implications for future research. | 9 |
| **FUNDING** | | |  |
| Funding | 27 | Describe sources of funding for the systematic review and other support (e.g., supply of data); role of funders for the systematic review. | 9 |

*From:*  Moher D, Liberati A, Tetzlaff J, Altman DG, The PRISMA Group (2009). Preferred Reporting Items for Systematic Reviews and Meta-Analyses: The PRISMA Statement. PLoS Med 6(7): e1000097. doi:10.1371/journal.pmed1000097.

| **Supplementary Table 2.** Electronic search strategy for the meta-analysis conducted through March 2020 | |
| --- | --- |
| Database; search | Search Terms |
| PubMed;  *k = 1152* | ((("Solitary confinement"[Title/Abstract] OR segregation[Title/Abstract] OR seclusion[Title/Abstract] OR "administrative segregation"[Title/Abstract] OR “disciplinary segregation”[Title/Abstract] OR “disciplinary confinement”[Title/Abstract] OR “punitive segregation”[Title/Abstract] OR "administrative confinement"[Title/Abstract] OR "protective segregation"[Title/Abstract] OR "protective custody"[Title/Abstract] OR "protective confinement"[Title/Abstract] OR "special housing unit"[Title/Abstract] OR "security housing unit"[Title/Abstract] OR "restrictive housing"[Title/Abstract] OR supermax[Title/Abstract] OR "maximum units"[Title/Abstract] OR "isolation units"[Title/Abstract]) AND (("mental health"[Title/Abstract] OR "psychological impact"[Title/Abstract] OR "psychological effect"[Title/Abstract] OR "behavioral impact"[Title/Abstract] OR "behavioral effect"[Title/Abstract] OR "medical impact"[Title/Abstract] OR "medical effect"[Title/Abstract] OR “psychological functioning”[Title/Abstract] OR “mental functioning”[Title/Abstract] OR “psychosocial functioning”[Title/Abstract] OR Depression[Title/Abstract] OR anxiety[Title/Abstract] OR loneliness[Title/Abstract] OR hallucinations[Title/Abstract] OR anger[Title/Abstract] OR aggressivity[Title/Abstract] OR irritability[Title/Abstract] OR violent[Title/Abstract] OR violence[Title/Abstract] OR nervousness[Title/Abstract] OR headaches[Title/Abstract] OR lethargy[Title/Abstract] OR tiredness[Title/Abstract] OR "Trouble sleeping"[Title/Abstract] OR insomnia[Title/Abstract] OR "heart palpitations"[Title/Abstract] OR "loss of appetite"[Title/Abstract] OR dizziness[Title/Abstract] OR nightmares[Title/Abstract] OR tremors[Title/Abstract] OR fainting[Title/Abstract] OR ruminations[Title/Abstract] OR "social withdrawal"[Title/Abstract] OR alienation[Title/Abstract] OR mood[Title/Abstract] OR suicide[Title/Abstract] OR mortality[Title/Abstract] OR Oversensitivity[Title/Abstract] OR hypersensivity[Title/Abstract]))) |
| PsycINFO;  *k = 1336* | (Abstract:("solitary confinement")) OR (Abstract:("disciplinary confinement")) OR (Abstract:("disciplinary segregation")) OR (Abstract:("punitive segregation")) OR (Abstract:(segregation)) OR (Abstract:(seclusion)) OR (Abstract:("administrative segregation")) OR (Abstract:("administrative confinement")) OR (Abstract:("protective segregation")) OR (Abstract:("protective custody")) OR (Abstract:("protective confinement")) OR (Abstract:("special housing unit")) OR (Abstract:("secure housing unit")) OR (Abstract:("restrictive housing")) OR (:(supermax)) OR (:("maximum units")) OR (:("isolation units"))) AND ( ((Abstract:("Physiological effect")) OR (Abstract:("Physiological impact")) OR (Abstract:("mental health")) OR (Abstract:("psychological impact")) OR (Abstract:("psychological functioning")) OR (Abstract:("psychosocial functioning")) OR (Abstract:("mental functioning")) OR (Abstract:("psychological effect")) OR (Abstract:("behavioral impact")) OR (Abstract:("behavioral effect")) OR (Abstract:("medical impact")) OR (Abstract:("medical effect")) OR (Abstract:(anxiety)) OR (Abstract:(depression)) OR (Abstract:(loneliness)) OR (Abstract:(hallucinations)) OR (Abstract:(anger)) OR (Abstract:(aggressivity)) OR (Abstract:(irritability)) OR (Abstract:(violent)) OR (Abstract:(violence)) OR (Abstract:(nervousness)) OR (Abstract:(headaches)) OR (Abstract:(lethargy)) OR (Abstract:(tiredness)) OR (Abstract:("Trouble sleeping")) OR (Abstract:(insomnia)) OR (Abstract:("heart palpitations")) OR (Abstract:("loss of appetite")) OR (Abstract:(dizziness)) OR (Abstract:(nightmares)) OR (Abstract:(tremors)) OR (Abstract:(fainting)) OR (Abstract:(ruminations)) OR (Abstract:("social withdrawal")) OR (Abstract:(alienation)) OR (Abstract:(mood)) OR (Abstract:(suicide)) OR (Abstract:(mortality)) OR (Abstract:(oversensitivty)) OR (Abstract:(hypersensitivity)) OR *same terms in Title.* |
| Web of Science;  *k = 661* | (TI=(“Solitary confinement” OR segregation OR seclusion OR “administrative segregation” OR “administrative confinement” OR “disciplinary segregation” OR “disciplinary confinement” OR “punitive segregation” OR “protective segregation” OR “protective custody” OR “protective confinement” OR “special housing unit” OR “restrictive housing” OR supermax OR “maximum units” OR “isolation units” )) AND (TS=(“Physiological effect” OR “physiological impact” OR “mental health” OR “psychological functioning” OR “psychosocial functioning” OR “mental functioning” OR “psychological impact” OR “psychological effect” OR “behavioral impact” OR “behavioral effect” OR “medical impact” OR “medical effect” OR Depression OR anxiety OR loneliness OR hallucinations OR anger OR aggressivity OR irritability OR violent OR violence OR nervousness OR Headaches OR Lethargy OR tiredness OR “Trouble sleeping” OR insomnia OR “Heart palpitations” OR “Loss of appetite” OR Dizziness OR Nightmares OR tremors OR fainting OR ruminations OR “social withdrawal” OR alienation OR mood OR suicide OR mortality OR Oversensitivity OR hypersensivity)) |
| Notes: 1. French translation of all terms were also included; 2. A search in **Google Scholar and bibliographies** enabled the identification of additional k=12 studies. | |

| **Supplementary Table 3**. Details of retrieved studies included in the meta-analysis and/or systematic review | | | | | | | | |
| --- | --- | --- | --- | --- | --- | --- | --- | --- |
|  |  |  |  | Quality of evidence | | | | |
| Author, year | Country | Type of SC | Sub-analyses | Study design^a^ | Controlled factors^a^ | Outcome measures | Total sample | Study quality |
| Chadick et al., 2018 | U.S | Administrative | Mood  Psychotic | Longitudinal | Age, ethnicity, time in incarceration, psychiatric diagnosis, most severe crime, pre-test scores. | Self-report: *Millon Clinical Multiaxial Inventory-III* | 48 | Moderate |
| Miller et Young, 1997 | U.S | Administrative/Disciplinary | Mood  Psychotic  Hostility  /Aggressivity | Cross-  sectional | Age and ethnicity | Self-report: *Brief Symptom Inventory* (BSI) | 30 | Low |
| O’Keefe, 2007 | U.S | Mixed | Mood  Psychotic  Hostility  /Aggressivity | RetrospectiveLongitudinal | - | Routine clinical evaluation: *Brief Psychiatric Rating Scale* (BPRS) | 4 245 | Moderate |
| O’Keefe et al., 2010 | U.S | Administrative/Disciplinary | Mood  Psychotic  Hostility  /Aggressivity | Cross-sectional | - | Trained interviewer: BPRS | 134 | Low |
| Zinger and Wichmann, 1999 | CAN | Administrative/Protective | Mood  Hostility  /Aggressivity | Longitudinal | - | Trained interviewers: BSI  *Beck*  *Depression;*  *Hopelessness*  *Scale;*  *State-Trait*  *Anxiety*  *Inventory;*  *Aggression*  *Questio-nnaire* | 60 | Moderate |
| Wildeman and Andersen, 2020 | DEN | Administrative/Disciplinary | Mortality | RetrospectiveLongitudinal | Age, ethnicity, sex, time in incarceration, type of crime, release year, education, other disciplinary actions, security-level and time segregated. | Mortality by all and unnatural (accidents, self-harm and violence) causes | 13 776 | Moderate |
| Brinkley-Rubinstein et al., 2019 | U.S | Mixed | Mortality | RetrospectiveLongitudinal | Age, sex, ethnicity, time in incarceration, violence- or drug-related convictions, mental health | Mortality by all causes, opioid overdose, homicide and suicide. | 229 274 | High |
|  |  |  |  |  | screening recommended or treatment and prior incarcerations. |  |  |  |
| Studies included exclusively in systematic review | | | | | | | | |
| Andersen et al., 2003 | DEN | Mixed | Mood | Longitudinal | - | Clinical evaluation: *Hamilton Anxiety and Depression Scales* | 119 | Moderate |
| Hagan et al., 2018 | U.S | Mixed | Mood | RetrospectiveLongitudinal | Age, gender, time in incarceration, time from release, present substance use, history of alcohol use disorder and parole status. | Clinical evaluation; *PC-PTSD* | 119 | Moderate |
| Kaba et al., 2014 | U.S | Disciplinary | Aggressivity (self-harm) | Retrospective | Age, ethnicity, time in incarceration and severe mental illness. | Clinical evaluation: self-harm reports | 134 188 | Moderate |
| Lanes et al., 2009 | U.S | Administrative | Aggressivity (self-harm) | Retrospective | Age, time in incarceration, education, mood disorder, having a major medical | Clinical evaluation: self-harm reports | 264 | Moderate |
|  |  |  |  |  | condition, history of suicide attempt, history of mental health contact, prior violent offenses, custody level, prior misconduct, prior housing/lock moves, prior protective custody |  |  |  |
| Reiter et al., 2020 | U.S | Disciplinary | Mood  Psychotic  Hostility  /Aggressivity | RetrospectiveLongitudinal | - | Semi-structured interview: BPRS | 16 571 | Moderate |
| Suedfeld et al., 1982 | U.S and  CAN | Disciplinary/  Protective | Mood  Hostility  /Aggressivity | Cross-sectional | - | Self-report: *The Multiple Affect Adjective Checklist (MAACL)* | 103 | Low |
| Mixed: could include all three forms of SC, i.e. administrative, disciplinary and protective. ^a^ Based-on outcome measure used specifically | | | | | | | | |
